# Supplementary material for: Regional Temperature-Sensitive Diseases and Attributable Fractions in China
Source: Int J Environ Res Public Health. 2019 Dec 26;17(1):184. doi: 10.3390/ijerph17010184 (PMC6982219; doi:10.3390/ijerph17010184)
Supplement: Supplementary file 1 [file ijerph-17-00184-s001.pdf]

## Supplementary

**Table S1.** The overall CRR of daily total mortality associated with extreme temperatures, using alternative maximum lags.

| Total | MMT | Extreme hot | Extreme cold |
|---|---|---|---|
| 30d | 25.4 | 1.13(1.09,1.18) | 1.30（1.10,1.54) |
| 21d | 24.1 | 1.13（1.09,1.18) | 1.30（1.15,1.47) |
| 14d | 25.5 | 1.13（1.09,1.18) | 1.31（1.19,1.45) |
| 7d | 23.9 | 1.14（1.09,1.18) | 1.19（1.09,1.29) |

**Table S2.** The overall CRR of daily total mortality associated with extreme temperatures, with and without adjustment of air pollutants.

| Adjustm Pollutions | Mortality | MMT | CRR | |
|---|---|---|---|---|
| | | | Extreme Heat | Extreme Cold |
| | total | 24.6 | 1.16(1.12,1.21) | 1.29（1.11,1.51) |
| without | circulatory system diseases | 23.1 | 1.23(1.17,1.30) | 1.45(1.17,1.80) |
| | Diseases of respiratory system | 23.3 | 1.26（1.18,1.35) | 1.30（1.03,1.65) |
| | total | 25.4 | 1.13(1.09,1.18) | 1.30(1.10,1.54) |
| with lag0 | circulatory system diseases | 23.2 | 1.19(1.12,1.26) | 1.46(1.16,1.82) |
| | Diseases of respiratory system | 22.4 | 1.25(1.09,1.30) | 1.34(1.07,1.42) |
| | total | 24.8 | 1.14(1.09,1.18) | 1.30(1.08,1.52) |
| with lag2 | circulatory system diseases | 23.1 | 1.19(1.12,1.26) | 1.46(1.16,1.82) |
| | Diseases of respiratory system | 21.8 | 1.23(1.12,1.35) | 1.23(1.02,1.48) |

The adjustment of the-day concentrations of fine particulate matter and ozone, which were conducted in 17 study sites with 3-year data

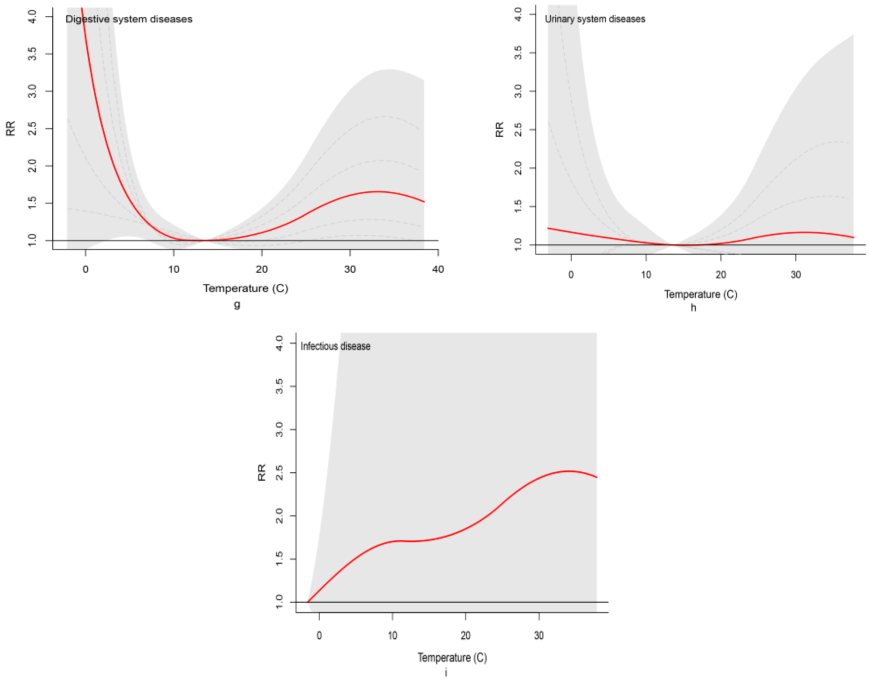


**Figure S1.** Overall exposure–response relationship between daily maximum temperature and cause-specific mortality by 30 d lag at 17 study sites in China (g-i): urinary system, digestive system and infectious diseases.
